# Clinical outcomes of trifluridine/tipiracil plus bevacizumab versus trifluridine/tipiracil or regorafenib in metastatic colorectal cancer: a multicenter cohort study

**DOI:** 10.1016/j.esmoop.2026.106907

**Published:** 2026-03-26

**Authors:** F. Moik, F. Huemer, B. Doleschal, H. Taghizadeh, P. Reimann, S.M. Kostmann, L. Wagner, M. Abdel Hamid, K.C. Zimmer, S.A. Suppé, C. Bachleitner, R. Schaberl-Moser, A. Amann, D. Wolf, R. Greil, P.J. Jost, T. Winder, H. Rumpold, L. Weiss, A. Gerger, A. Seeber, J.M. Riedl

**Affiliations:** 1 Division of Oncology, Department of Internal Medicine, Medical University of Graz, Graz, Austria; 2 Department of Internal Medicine III with Haematology, Medical Oncology, Haemostaseology, Infectiology and Rheumatology, Oncologic Center, Paracelsus Medical University Salzburg, Salzburg, Austria; 3 Department of Internal Medicine I for Hematology With Stem Cell Transplantation, Hemostaseology and Medical Oncology, Ordensklinikum Linz, Linz, Oberösterreich; 4 Department of Internal Medicine, Universitätsklinikum St. Pölten, Karl Landsteiner University of Health Sciences, St. Pölten, Austria; 5 Department of Internal Medicine II, Academic Teaching Hospital Feldkirch, Feldkirch, Austria; 6 Department of Internal Medicine V (Hematology and Oncology), Comprehensive Cancer Center Innsbruck, Medical University of Innsbruck, Innsbruck, Austria; 7 BioTechMed-Graz, Graz, Austria; 8 Austrian Breast and Colorectal Cancer Study Group (ABCSG), Vienna, Austria; 9 Department of Oncology, Hematology and Palliative Care, General Hospital of Oberwart, Oberwart, Austria; 10 Massachusetts General Hospital Cancer Center and Department of Medicine, Harvard Medical School, Boston, USA

**Keywords:** metastatic colorectal cancer, trifluridine/tipiracil + bevacizumab, FTD–TPI, regorafenib, *KRAS*

## Abstract

**Background:**

In patients with pretreated metastatic colorectal cancer (mCRC), trifluridine/tipiracil (FTD–TPI) + bevacizumab has superior efficacy compared with FTD–TPI alone. However, real-world data on outcomes and comparisons with other established therapies including regorafenib are scarce. Additionally, evidence suggests that molecular alterations, such as *KRAS*^*G12*^ mutations, may affect FTD–TPI efficacy. We aimed to compare clinical outcomes of patients with mCRC treated with FTD–TPI + bevacizumab, FTD–TPI, or regorafenib across molecular subgroups.

**Patients and methods:**

This retrospective cohort study included 509 patients treated at six Austrian cancer centers between January 2015 and December 2022. The primary outcome was progression-free survival (PFS), with overall survival (OS) and disease control rate (DCR) as secondary outcomes. Between-group differences were analyzed using Cox and logistic regression. A propensity score analysis using inverse probability of treatment weighting (IPTW) was conducted. Flexible parametric models and restricted mean survival time (RMST) analyses were used to account for time-dependent differences in PFS/OS.

**Results:**

In propensity score analysis, patients treated with FTD–TPI + bevacizumab (*n* = 130) versus FTD–TPI (*n* = 227) alone or regorafenib (*n* = 152) had longer PFS [IPTW-adjusted hazard ratio (HR) 0.68, 95% confidence interval (CI) 0.51-0.92, *P* = 0.012] and higher DCR (IPTW-adjusted odds ratio 3.35, 95% CI 1.81-6.20, *P* < 0.001). The IPTW-adjusted HR for OS was 0.80 (95% CI 0.58-1.12, *P* = 0.203). A modest OS benefit of FTD–TPI + bevacizumab was observed within the first 12 months. In RMST-analysis, OS was 12.1 months (95% CI 11.4-12.8 months) for FTD–TPI + bevacizumab compared with 10.6 months (95% CI 9.9-11.2 months) for the controls (*P* = 0.003). Outcomes were consistent across key molecular subsets, including *KRAS* and *KRAS*^*G12*^ mutations.

**Conclusions:**

In this large real-world cohort of patients with mCRC, FTD–TPI + bevacizumab was associated with improved PFS and DCR compared with FTD–TPI or regorafenib, with consistent benefit across most molecular subgroups, including *KRAS*^*G12*^ mutations.

## Highlights

• Real-world multicenter cohort study comparing FTD–TPI + bevacizumab versus FTD–TPI or regorafenib in pretreated mCRC.
• FTD–TPI + bevacizumab demonstrates superior real-world effectiveness.
• Benefit was consistent across key molecular subgroups including patients with *KRAS* and *KRAS*^*G12*^ mutations.

## Introduction

Colorectal cancer (CRC) represents the second leading cause of cancer-related death in the Western world.^1^ Improvements in the management of CRC have led to a constant decrease in mortality rates over the past decades.^1^ However, in patients with metastatic CRC (mCRC), the overall prognosis remains poor, with 5-year survival estimates of only around 10%.^1^

In recent years, the therapeutic resources for patients with mCRC has grown substantially with the implementation of combination treatment strategies, targeted therapies and immunotherapy for selected subsets of patients, and tailored continuous treatment approaches over multiple lines of palliative therapy.^2^ Further, late-line therapeutic options in patients with mCRC are expanding, with an increasing proportion of patients amenable for multiple lines of systemic therapies.^2^^,^^3^

Beyond disease progression for initial palliative systemic therapies, which typically comprise 5-flurouracil based combinations with irinotecan, oxaliplatin, and targeted therapies depending on mutational status, different systemic treatment options are currently available.^2^^,^^4^ In a small subset of patients, clinically actionable molecular alterations are detected that impact treatment options in the late-line therapeutic setting.^2^ In biomarker unselected patients who are refractory to established early treatment options, three main systemic treatment approaches with different toxicity profiles are currently available.^5^^,^^6^ Firstly, regorafenib led to improved outcomes as compared with placebo in patients with treatment-refractory mCRC in the CORRECT and CONCUR trials.^7^^,^^8^ Subsequently, the RECOURSE and TERRA trials showed a survival benefit with FTD–TPI over placebo in treatment-refractory mCRC.^9^^,^^10^ More recently, the SUNLIGHT trial reported that the combination of FTD–TPI and bevacizumab further improved survival compared with FTD–TPI monotherapy, establishing a new standard of care for mCRC all comer patients in third- and later-line treatments.^11^

However, real-world data on clinical outcomes with established, late-line therapies in biomarker unselected mCRC remain limited, and comprehensive comparative analyses are lacking. Additionally, emerging evidence suggests that the efficacy of FTD–TPI-based regimens may vary according to underlying molecular alterations, particularly *KRAS*^*G12*^ mutations.^12^ To address these gaps, we conducted a multicenter cohort study of patients with mCRC initiating treatment with FTD–TPI plus bevacizumab, FTD–TPI alone, or regorafenib with the aim to (i) provide real-world data on clinical outcomes of patients treated with established late-line therapies, (ii) assess comparative effectiveness across treatment groups, and (iii) explore response patterns in relation to molecular subtypes.

## Patients and methods

### Study population and design

In this multicenter, observational cohort study, all consecutive patients with mCRC treated with either FTD–TPI + bevacizumab, FTD–TPI monotherapy, or regorafenib between 1 January 2015 until 31 December 2022 from six cancer centers in Austria were included. Patient data were obtained by individual chart review. Patient- and disease-specific characteristics were obtained at study baseline, with the index date defined as the date of initiation of the respective palliative treatment of interest. Baseline covariables include patient age, sex, body mass index (BMI), Charlson comorbidity index (CCI), Eastern Cooperative Oncology Group (ECOG) performance status, cancer and treatment specifics including sidedness, metastatic patterns, mutational status, line of palliative treatment, and laboratory values including levels of carcinoembryonic antigen (CEA).

Patients were followed from the day of treatment initiation (index date) until the occurrence of an outcome event, death, or loss of follow-up, censoring patients at the last known date without the respective outcome of interest. The main study outcome was progression-free survival (PFS), defined as the time until either radiologic disease progression or death.^13^ Secondary outcomes were disease control rate (DCR), defined as the investigator-assessed composite of complete response, partial response, or stable disease in analogy to RECIST v1.1; overall response rate (ORR), defined as the composite of complete or partial response; and overall survival (OS), defined as the time from treatment initiation to death from any cause.

The study was approved by the institutional review board of the coordinating center (ethics committee of the Medical University of Graz, Austria; approval number 35-486 ex 22/23). All methods were carried out in accordance with relevant local and national guidelines and regulations.

### Statistical analysis

Continuous variables were summarized using medians and corresponding interquartile range (IQR), and count data using absolute frequencies (%). Between-group differences in baseline covariables were assessed using the Kruskal–Wallis test for continuous, non-parametric data, and chi-square or Fisher´s exact tests were used for frequencies, as appropriate. The reverse Kaplan–Meier method was used to estimate available follow-up time. Kaplan–Meier analysis was used to obtain unadjusted survival estimates and corresponding 95% confidence intervals (95% CI) for PFS and OS, using log-rank tests for between-group comparisons. For uni- and multivariable modeling of time-to-event in PFS and OS analysis, Cox proportional hazards models were fitted, obtaining hazard ratios (HR) and corresponding 95% CI, whereas the association of treatment type with binary outcomes (DCR and ORR) was analyzed in a logistic regression model, obtaining odds ratios (OR) and corresponding 95% CI.

A propensity score was calculated to predict the probability of treatment assignment conditional on baseline covariates.^14^, ^15^, ^16^ The propensity score was calculated using a multivariable logistic regression model, including a broad range of baseline clinicopathologic covariables, irrespective of their association with outcome variables, as reported in Supplementary Table S1, available at https://doi.org/10.1016/j.esmoop.2026.106907.^14^ For this model, missing baseline covariates were imputed using a chained equations algorithm with 25 imputation datasets.^17^ A list of variables used for multiple imputation is provided in Supplementary Table S2, available at https://doi.org/10.1016/j.esmoop.2026.106907. Propensity scores were estimated within each imputed dataset using the same model specification and pooled using Rubin’s rules, without additional standardization or transformation of continuous variables beyond imputation. Subsequently, the propensity score was used to calculate the individual inverse probability of treatment weight (IPTW), as the inverse of the probability of receiving the treatment an individual patient actually did receive. Re-weighting of data was used to control for between-group imbalances in outcome analyses. Covariate balance upon IPTW-adjustment was assessed by quantifying the standardized mean differences (SMD) in baseline covariables between treatment groups, with SMDs >0.20 considered to indicate potentially relevant imbalance between the study groups.^14^ The proportional hazards assumption was evaluated by visual inspection of survival estimates and hazard curves, and by Schoenfeld test *P* values. A flexible parametric model was used to explore potential time-dependencies in between-group differences. Here, an IPTW-weighted survival model with restricted cubic splines on the log (cumulative hazard) scale (3 degrees of freedom for the time-invariant and 2 degrees of freedom for the time-dependent between-group differences) was used. To further account for potential violations of the proportional hazards assumption, restricted mean survival time (RMST) analyses were conducted for PFS and OS, calculating the area under the Kaplan–Meier curve across the 24-month follow-up period. Here, RMSTs were obtained for treatment groups, calculating the relative efficacy gain as the absolute difference in RMST divided by the RMST in the control group.^18^^,^^19^ Subgroup analyses according to mutational status were carried out. All statistical analyses were carried out using Stata (Windows version 15.1, Stata Corp., Houston, TX, USA).

## Results

### Study cohort

Overall, 509 patients with mCRC undergoing palliative treatment with FTD–TPI + bevacizumab, FTD–TPI alone, or regorafenib were included in the present study. Median age at treatment initiation was 66 years (IQR 58-73 years), and 307 patients were male (60.3%). The median CCI was 3 (IQR 2-4); 202 patients (43.5%) had an ECOG performance status of 0, 216 patients (46.6%) an ECOG 1 and 46 patients (9.9%) of ≥2.

A right-sided primary tumor was diagnosed in 137 patients (26.9%) and 271 patients (53.4%) had primary metastatic disease at initial diagnosis. The most common metastatic sites were liver (*n* = 382, 75.1%), lung (*n* = 320, 62.9%), and peritoneum (*n* = 121, 23.8%). *RAS* mutations were detected in 296 patients (58.6%), including *KRAS*^*G12*^ mutations in 154 patients (30.5%).

Overall, 130 patients were treated with FTD–TPI + bevacizumab (25.5%), 227 with FTD–TPI (44.6%), and 152 with regorafenib (29.9%). Treatment was most commonly initiated as third-line palliative therapy (median line 3; IQR 2-4). Significant between-group differences were observed according to patient age, comorbidity burden, ECOG performance status, and palliative treatment line. No significant differences were observed in the prevalence of *RAS* mutations, yet the distribution of *KRAS*^*G12*^ mutations varied between groups. No significant between-group differences were observed in respect to sex, BMI, cancer sidedness, metastatic pattern, and levels of CEA. Detailed cohort characteristics including information on the overall study cohort and stratified by treatment type are provided in Table 1.

**Table 1:** Study cohort characteristics

Variable	Missing *n* (%)	Overall cohort (*n* = 509)	FTD–TPI + bevacizumab (*n* = 130)	FTD–TPI (*n* = 227)	Regorafenib (*n* = 152)	*P*^a^
Age	0 (0%)	65.9 (58.2-73.4)	67 (59-76)	68 (59-76)	63 (56-69)	<0.001
Male sex	0 (0%)	307 (60.3%)	73 (57.0%)	133 (58.9%)	101 (66.5%)	0.207
BMI	91 (17.8%)	24.4 (21.5-27.4)	23.8 (21.1-26.9)	24.3 (21.5-27.7)	24.6 (22.1-27.5)	0.321
CCI	99 (19.4%)	3 (2-4)	3 (2-4)	3 (2-4)	2 (1-4)	0.005
CCI 0-1	–	87 (21.2%)	28 (22.4%)	32 (17.0%)	27 (27.8%)	0.067
CCI 2-3	–	180 (43.9%)	58 (46.4%)	78 (41.5%)	44 (45.4%)	–
CCI ≥4	–	143 (34.9%)	39 (31.2%)	78 (41.5%)	26 (26.8%)	–
ECOG PS	7 (1.4%)	–	–	–	–	0.02
0	–	202 (43.5%)	54 (47.8%)	75 (36.4%)	73 (50.3%)	–
1	–	216 (46.6%)	51 (45.1%)	102 (49.5%)	63 (43.5%)	–
2-3	–	46 (9.9%)	8 (7.1%)	29 (14.1%)	9 (6.2%)	–
Right-sided primary	0 (0%)	137 (26.9%)	41 (31.5%)	55 (24.3%)	41 (27.0%)	0.325
Primary metastatic at diagnosis (synchronous)	1 (0.2%)	271 (53.4%)	63 (48.8%)	109 (48.0%)	65 (42.8%)	0.511
Liver metastasis	0 (0%)	382 (75.1%)	85 (65.4%)	138 (60.8%)	97 (63.8%)	0.660
Lung metastasis	0 (0%)	320 (62.9%)	97 (74.6%)	163 (71.8%)	122 (80.3%)	0.174
Peritoneal metastasis	0 (0%)	121 (23.8%)	29 (22.3%)	55 (24.2%)	37 (24.3%)	0.902
*RAS* mutated	4 (0.7%)	296 (58.6%)	79 (61.7%)	128 (56.9%)	89 (58.6%)	0.675
*KRAS* mutated	4 (0.7%)	276 (54.7%)	71 (59.2%)	126 (56.5%)	79 (55.6%)	0.836
*KRAS*^*G12*^ mutated	4 (0.7%)	154 (30.5%)	50 (39.1%)	69 (30.7%)	35 (23.0%)	0.015
*NRAS* mutated	4 (0.7%)	20 (4.0%)	8 (14.0%)	2 (2.0%)	10 (13.7%)	0.007
*PIK3CA* mutated	339 (66.6%)	34 (20.0%)	14 (23.3%)	17 (21.8%)	3 (9.4%)	0.243
*TP53* mutated	366 (71.9%)	85 (59.4%)	31 (72.1%)	39 (54.9%)	15 (51.7%)	0.124
*BRAF*^*V600E*^ mutated	88 (17.3%)	22 (5.2%)	8 (6.7%)	10 (5.1%)	4 (3.8%)	0.627
Treatment line	0 (0%)	3 (3-4)	3 (2-3)	3 (3-4)	3 (3-4)	<0.001
Prior systemic treatment lines	0 (0%)	–	–	–	–	<0.001
≤1	–	120 (23.6%)	43 (33.1%)	55 (24.2%)	22 (14.5%)	–
2	–	245 (48.1%)	67 (51.5%)	101 (44.5%)	77 (50.7%)	–
3	–	100 (19.7%)	14 (10.8%)	52 (22.9%)	34 (22.4%)	–
≥4	/	44 (8.6%)	6 (4.6%)	19 (8.4%)	19 (12.5%)	/
Prior systemic therapy	0 (0%)	–	–	–	–	–
Fluoropyrimidine	–	483 (94.9%)	117 (90.0%)	218 (96.0%)	148 (97.4%)	0.011
Oxaliplatin	–	337 (66.2%)	69 (53.1%)	155 (68.3%)	113 (74.3%)	0.001
Irinotecan	–	398 (78.2%)	88 (67.7%)	171 (75.3%)	139 (91.5%)	<0.001
VEGF-antibody	–	407 (80.0%)	103 (79.2%)	171 (75.3%)	133 (87.5%)	0.014
EGFR-antibody	–	170 (33.4%)	42 (32.3%)	78 (34.4%)	50 (32.9%)	0.913
CEA levels, μg/l	326 (36.0%)	52.8 (10.9-242.0)	58.2 (15.1-192.0)	64.8 (11.1-254.4)	41.1 (9.0-170-0)	0.708
Subsequent systemic therapy	0 (0%)	167 (32.8%)	39 (30.0%)	77 (33.9%)	51 (33.6%)	0.316
1 further line	–	106 (20.8%)	22 (16.9%)	52 (22.9%)	32 (21.1%)	–
≥2 further lines	–	62 (12.2%)	17 (13.1%)	25 (11.0%)	19 (12.5%)	–
Subsequent FTD–TPI	–	69 (13.6%)	0	0	69 (45.4%)	–
Subsequent FTD–TPI + bevacizumab	–	3 (0.6%)	0	0	3 (2.0%)	–
Subsequent regorafenib	–	101 (19.8%)	39 (30.0%)	62 (27.3%)	0	–

BMI, body mass index; CCI, Charlson comorbidity index; CEA, carcinoembryonic antigen; ECOG PS, Eastern Cooperative Oncology Group performance status; EGFR, endothelial growth factor receptor; FTD–TPI, trifluridine/tipiracil; VEGF, vascular endothelial growth factor.

a Kruskal–Wallis test for continuous, non-parametric data; chi-Square or Fisher´s exact test for frequencies, as appropriate.

### Unadjusted analysis of treatment: response and survival outcomes

Over a median follow-up of 24.0 months, 415 deaths (81.5% of the study cohort) and 435 disease progression events (85.5%) were observed.

The overall DCR and ORR were 30.1% and 6.1%, respectively. The DCR was statistically significantly higher in patients treated with FTD–TPI + bevacizumab (51.3%), compared with those treated with FTD–TPI (22.6%) or regorafenib (23.6%) (chi-square *P* < 0.001). The ORR was 9.7% for FTD–TPI + bevacizumab, 5.1% for FTD–TPI, and 4.7% for regorafenib (chi-square *P* = 0.180).

The median PFS in the overall cohort was 3.2 months (95% CI 3.0-3.4 months), with corresponding cumulative PFS estimates at 6, 12, and 24 months of 22.0% (95% CI 18.3% to 25.9%), 5.9% (95% CI 3.9% to 8.5%) and 1.7% (95% CI 0.1% to 3.6%), respectively. In the unadjusted analysis, PFS was significantly longer in patients treated with FTD–TPI + bevacizumab (median PFS 4.1 months; 95% CI 3.5-5.3 months) compared with those receiving FTD–TPI alone (3.0 months; 95% CI 2.7-3.2 months) or regorafenib (3.0 months; 95% CI 2.5-3.4 months) (log-rank *P* < 0.001).

**Figure 1:**
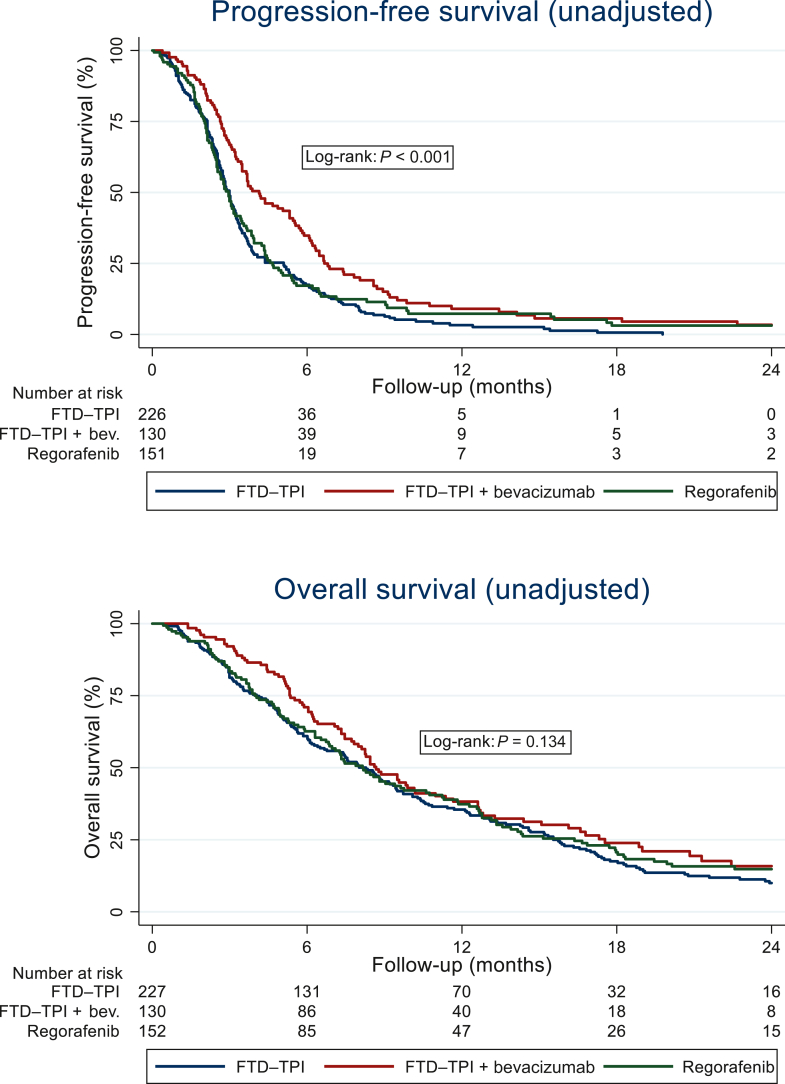
**Unadjusted Kaplan–Meier estimates of PFS and OS.** bev., bevacizumab; FTD–TPI, trifluridine/tipiracil; OS, overall survival; PFS, progression-free survival.

The median OS after treatment initiation was 8.4 months (95% CI 7.5-9.2 months), with cumulative OS estimates at 6, 12, and 24 months of 64.0% (95% CI 59.6% to 68.1%), 36.7% (95% CI 32.3% to 41.1%) and 13.4% (95% CI 10.3% to 17.0%), respectively. OS did not differ significantly between patients receiving FTD–TPI + bevacizumab and those treated with FTD–TPI or regorafenib (log-rank *P* = 0.134). Median OS was 8.7 months (95% CI 7.7-10.2 months), 8.3 months (95% CI 6.5-9.4 months), and 8.1 months (95% CI 6.8-10.6 months), respectively. In Figure 1, unadjusted Kaplan–Meier estimates of PFS and OS for patients treated with FTD–TPI + bevacizumab, FTD–TPI, and regorafenib are displayed. In Table 2, detailed treatment response and survival data of patients overall and stratified by treatment group are summarized.

**Table 2:** Clinical outcomes in unadjusted analysis

	Analysis cohort^a^	Overall	FTD–TPI + bevacizumab	FTD–TPI	Regorafenib	*P*^c^
DCR, *n* (%)	448 (88.0%)	135 (30.1%)	58 (51.3%)	48 (22.6%)	29 (23.6%)	<0.001
ORR, *n* (%)	457 (89.8%)	28 (6.1%)	11 (9.7%)	11 (5.1%)	6 (4.7%)	0.180
PFS^b^, median (95% CI)	509 (100%)	3.2 (3.0-3.4)	4.1 (3.5-5.3)	3.0 (2.7-3.2)	3.0 (2.5-3.4)	<0.001
6 months	–	22.0% (18.3-25.9)	34.8% (26.3-43.4)	17.5% (12.7-22.9)	17.2% (10.9-24.6)	–
12 months	–	5.6% (3.6-8.2)	9.0% (4.5-15.5)	2.6% (0.9-5.9)	7.3% (3.3-13.3)	–
24 months	–	1.7% (0.7-3.6)	3.4% (1.0-8.6)	0%	3.1% (0.9-8.0)	–
OS^b^, median (95% CI)	509 (100%)	8.4 (7.5-9.2)	8.7 (7.7-10.2)	8.3 (6.5-9.4)	8.1 (6.8-10.6)	0.134
6 months	–	64.0% (59.6-68.1)	71.0% (62.1-78.1)	61.0% (54.2-67.1)	62.6% (54.1-70.0)	–
12 months	–	36.7% (32.3-41.1)	38.2% (29.4-47.0)	35.5% (29.1-41.9)	37.3% (29.2-45.5)	–
24 months	–	12.8% (9.7-16.3)	15.9% (8.8-24.7)	10.0% (6.2-14.8)	14.8% (9.2-21.6)	–

CI, confidence interval; DCR, disease control rate; FTD–TPI, trifluridine/tipiracil; ORR, overall response rate; OS, overall survival.

a DCR and ORR only evaluable in patients who underwent follow-up radiologic restaging procedures.

b Kaplan–Meier estimates in months.

c *P* values obtained via chi-square test for frequencies, and log-rank test for survival times.

### Derivation of the propensity score

To further explore the observed improved outcomes of patients treated with FTD–TPI + bevacizumab compared with those treated with single-agent FTD–TPI or regorafenib, a propensity score analysis was conducted. Differences in baseline characteristics between treatment groups suggested potential imbalances in underlying confounders (Table 1). To account for these differences, a propensity score was calculated to estimate the probability of treatment assignment based on relevant baseline covariates (Supplementary Figures S1 and S2, available at https://doi.org/10.1016/j.esmoop.2026.106907). Subsequently, the IPTW was derived, according to the inverse of the probability of receiving the treatment that the patient received. Adequate covariate balance was achieved upon IPTW-weighing of data, as indicated by a SMD of <0.2 for all variables included in the derivation of the propensity score (Supplementary Table S3, available at https://doi.org/10.1016/j.esmoop.2026.106907).

### IPTW-adjusted analysis of treatment: response and survival outcomes

**Figure 2:**
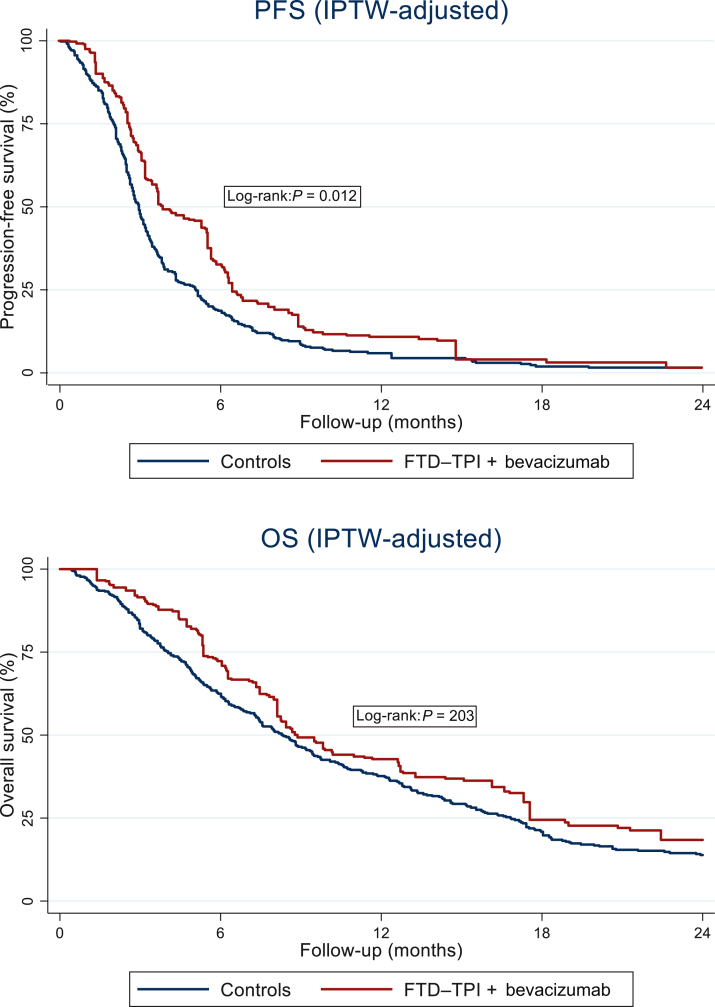
IPTW-adjusted PFS and OS estimates. FTD–TPI, trifluridine/tipiracil; IPTW, inverse probability of treatment weight; OS, overall survival; PFS, progression-free survival.

Treatment with FTD–TPI + bevacizumab was associated with longer PFS compared with control patients (IPTW-adjusted HR for PFS 0.68, 95% CI 0.51-0.92, *P* = 0.012). The median IPTW-adjusted PFS estimate was 3.7 months with FTD–TPI + bevacizumab, in contrast with 3.0 months in control patients. The PFS estimates at 3, 6, 12 and 18 months of follow-up in patients treated with FTD–TPI + bevacizumab were 62.5%, 28.5%, 7.7%, and 3.2%, respectively, compared with 48.4%, 17.5%, 5.2%, and 1.8% in control patients (Figure 2A, Supplementary Table S4, available at https://doi.org/10.1016/j.esmoop.2026.106907).

Upon IPTW-weighing, treatment with FTD–TPI + bevacizumab was associated with a higher DCR compared with control patients (IPTW-adjusted OR 3.35, 95% CI 1.81-6.20, *P* < 0.001). The IPTW-adjusted OR for ORR was 2.25, 95% CI 0.81-6.25, *P* = 0.123.

The IPTW-adjusted HR for OS was 0.80, 95% CI 0.58-1.12, *P* = 0.203). The adjusted median OS estimate was 8.7 months for patients treated with FTD–TPI + bevacizumab and 8.3 months for control patients (Figure 2B, Supplementary Table S4, available at https://doi.org/10.1016/j.esmoop.2026.106907). A proportion of patients crossed over to alternative treatments investigated in this study in subsequent lines of therapy (Table 1), which may have attenuated between-group differences in OS. In a sensitivity analysis censoring OS at initiation of subsequent systemic therapy conducted in patients with available data on subsequent treatment start date (*n* = 374), the magnitude of benefit associated with FTD–TPI + bevacizumab increased, with an IPTW-adjusted HR for OS of 0.66, 95% CI 0.41-1.07, *P* = 0.091).

Overall, similar results were obtained when restricting the control group to single-agent treatments (FTD–TPI + bevacizumab versus FTD–TPI or regorafenib alone; Supplementary Table S5, available at https://doi.org/10.1016/j.esmoop.2026.106907).

### Time-dependent differences in clinical outcomes between treatment groups

Graphical inspection of Kaplan–Meier estimates of PFS and OS curves indicated potential violation of the proportional hazard assumption. This was supported by formal testing using Schoenfeld residuals for PFS (*P* = 0.002) and for OS (*P* = 0.025). Thus, a flexible parametric survival model was used to explore potential time-dependent effects in PFS and OS comparisons between groups.

In the PFS analysis, control patients exhibited a higher rate of disease progression early during follow-up compared with those treated with FTD–TPI + bevacizumab, with progression rates crossing between 6 and 12 months of follow-up (Supplementary Figure S3, available at https://doi.org/10.1016/j.esmoop.2026.106907). After accounting for time-dependent effects in predicted PFS estimates, treatment with FTD–TPI + bevacizumab was associated with increased PFS (Wald test *P* = 0.001; Supplementary Figure S4, available at https://doi.org/10.1016/j.esmoop.2026.106907). In a subsequent IPTW-weighted non-parametric survival model incorporating restricted cubic splines, FTD–TPI + bevacizumab remained associated with superior PFS compared with control patients (HR 0.49, 95% CI 0.32-0.74).

Similarly, flexible parametric modeling of OS revealed higher early mortality in control patients compared with those treated with FTD–TPI + bevacizumab (Supplementary Figure S5, available at https://doi.org/10.1016/j.esmoop.2026.106907). A borderline significant OS benefit was observed with FTD–TPI + bevacizumab after accounting for time-dependent effects (Wald test *P* = 0.051; Supplementary Figure S6, available at https://doi.org/10.1016/j.esmoop.2026.106907). In IPTW-weighted non-parametric modeling with restricted cubic splines, the HR for OS for patients treated with FTD–TPI + bevacizumab compared with controls was 0.63 (95% CI 0.39-1.00).

In RMST-analysis, the 24-month RMST for PFS was 5.7 months (95% CI 5.3-6.2 months) for FTD–TPI + bevacizumab compared with 4.2 months for controls (95% CI 3.9-4.6 months). The difference in RMST was 1.5 months (95% CI 0.9-2.1 months, *P* < 0.001) in favor of FTD–TPI + bevacizumab, with an efficacy gain of 34.9%. The 24-month RMST for OS was 12.1 months (95% CI 11.4-12.8 months) for FTD–TPI + bevacizumab compared with 10.6 months (95% CI 9.9-11.2 months) for controls (difference 1.5 months, 95% CI 0.5-2.5 months, *P* = 0.003), with an efficacy gain of 14.3%.

### Molecular subgroup analysis

**Figure 3:**
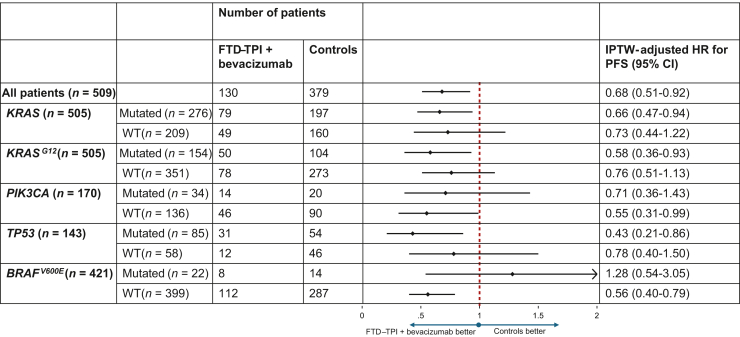
**Forest plot of IPTW-adjusted HRs for PFS (FTD–TPI + bevacizumab versus controls) according to molecular subgroups.** CI, confidence interval; FTD–TPI, trifluridine/tipiracil; HR, hazard ratio; IPTW, inverse probability of treatment weight; PFS, progression-free survival; WT, wild type.

To explore potential molecular determinants of treatment efficacy, clinical outcomes were stratified by mutational status. The PFS benefit of FTD–TPI + bevacizumab was consistent across key molecular subgroups, with IPTW-adjusted HRs of 0.66 (95% CI 0.47-0.94) in patients with *KRAS-*mutant and 0.73 (95% CI 0.44-1.22) in *KRAS* wild-type tumors. Comparable effects were also observed in patients with *KRAS*^*G12*^ mutations (HR 0.58, 95% CI 0.36-0.93) and *KRAS*^*G12*^ wild-type (HR 0.76, 95% CI 0.51-1.13). A significant PFS benefit was also seen in patients with *BRAF*^*V600E*^ wild-type tumors (HR 0.56, 95% CI 0.40-0.79), whereas no benefit was observed in those with *BRAF*^*V600E*^-mutations (HR 1.28, 95% CI 0.54-3.05). Exploratory interaction testing for PFS showed that treatment effects differed according to *BRAF*^*V600E*^ status (*P* = 0.020), whereas no significant interactions were observed for *KRAS*, *KRAS*^*G12*^, *TP53*, or *PIK3CA* status (all *P* ≥ 0.05). Detailed clinical outcomes including PFS, DCR, and OS according to molecular subgroups are presented in Figure 3 and Supplementary Table S6, available at https://doi.org/10.1016/j.esmoop.2026.106907, whereas Kaplan–Meier PFS estimates for the different treatment groups stratified by mutational status are shown in Supplementary Figure S7, available at https://doi.org/10.1016/j.esmoop.2026.106907.

## Discussion

In this retrospective, multicenter analysis of patients with mCRC, we comprehensively evaluated real-world outcomes associated with established late-line therapeutic options including FTD–TPI + bevacizumab, FTD–TPI monotherapy, and regorafenib.

Our findings indicate that combination treatment with FTD–TPI + bevacizumab is associated with improved clinical outcomes compared with FTD–TPI or regorafenib monotherapy. These associations remained robust after adjustment for potential confounding using propensity score weighting. Notably, a time-dependent treatment effect on PFS was observed, with the greatest benefit seen within the first 6 to 12 months of therapy. No statistically significant differences in OS were observed between treatment groups in unadjusted analysis, yet there were indications of a potential modest OS benefit observed within the first 12 months, as indicated by both RMST analysis and the time-dependent IPTW-weighted survival model, which account for a potential time-varying treatment effect over the 24-month follow-up period.

Notably, our subgroup analyses indicate a consistent benefit of FTD–TPI + bevacizumab compared with FTD–TPI and regorafenib across major molecular subgroups, including *KRAS*^*G12*^ mutations. Previously, *KRAS*^*G12*^ mutations—detected in 30.5% of our study cohort—were reported as a potential predictive biomarker for reduced benefit from FTD–TPI, based on experimental data and a *post hoc* subgroup analysis of the RECOURSE trial, comparing FTD–TPI with placebo.^12^ Conversely, a meta-analysis of three randomized controlled trials found no differential outcomes for FTD–TPI versus placebo according to *KRAS*^*G12*^ mutational status.^20^ Likewise, in the SUNLIGHT trial, which compared FTD–TPI plus bevacizumab with FTD–TPI monotherapy, *KRAS*^*G12*^ mutational status was not predictive of clinical outcomes,^21^ nor was it associated with OS in a real-world cohort study of patients with mCRC treated with FTD–TPI plus bevacizumab.^22^ Taken together, our findings, in line with previous evidence, provide compelling support that FTD–TPI + bevacizumab improves outcomes independently of KRAS mutational status.

Interestingly, the largest magnitude of PFS benefit for FTD–TPI + bevacizumab compared with those treated with FTD–TPI or regorafenib was seen in patients harboring a *TP53* mutation. This observation is intriguing and warrants further investigation, given previous studies suggesting a potential association between *TP53* loss-of-function mutations and improved clinical outcomes in patients treated with bevacizumab-containing regimens, both in mCRC and other tumor types.^23^, ^24^, ^25^, ^26^ Notably, no clinical benefit was observed with FTD–TPI + bevacizumab in patients harboring *BRAF*^*V600E*^ mutations, a patient subgroup that was significantly underrepresented in the phase III SUNLIGHT trial, and a trend toward inferior outcomes was noted. Although the limited subgroup sample size precludes definitive conclusions, these findings underline the aggressive nature of *BRAF*^*V600E*^-mutant mCRC and the unmet need for more effective therapeutic options following progression on *BRAF*^*V600E*^-targeted therapy.^27^^,^^28^

Our findings are in line with previous data indicating improved clinical outcomes upon combining FTD–TPI with bevacizumab in patients with pretreated mCRC. In detail, the observed DCR in the treatment groups in our study were slightly lower than the reported rates in the landmark phase III trials (Supplementary Table S7, available at https://doi.org/10.1016/j.esmoop.2026.106907).^7^^,^^9^^,^^11^ Further, PFS estimates for the FTD–TPI and regorafenib monotherapy arms in our study were comparable with those reported in clinical trials, whereas PFS for FTD–TPI + bevacizumab was shorter in our study than observed in the SUNLIGHT trial.^7^^,^^9^^,^^11^ Similarly, OS estimates in our study were comparable with those previously reported for FTD–TPI and regorafenib single-agent treatments, whereas OS estimates appeared slightly shorter for the FTD–TPI + bevacizumab combination compared with outcomes observed in the registrational phase III trial.^7^^,^^9^^,^^11^ Differences in key baseline characteristics between real-world and clinical trial populations may account for the observed discrepancy in OS outcomes.^29^ Compared with the SUNLIGHT trial, our real-world cohort included a less selected patient population, with a broader range of prior treatment lines. Approximately one-third of patients received FTD–TPI + bevacizumab in earlier lines than permitted in the SUNLIGHT trial. In addition, a subset of patients had incomplete prior exposure to standard agents and included individuals with ECOG performance status ≥2, potentially reflecting a more vulnerable population that may not have been eligible for established early-line combination chemotherapy regimens. These differences likely contribute to the moderately lower survival estimates observed in our cohort compared with the SUNLIGHT trial.

Importantly, our comparative analyses of efficacy outcomes support the clinical benefit of FTD–TPI + bevacizumab over FTD–TPI or regorafenib monotherapy in the real-world setting. Similar findings were reported in a real-world cohort study from Japan using an administrative database, showing that patients treated with FTD–TPI + bevacizumab had longer OS and time to treatment discontinuation compared with those treated with FTD–TPI or regorafenib monotherapies in a propensity score analysis.^30^ These consistent findings underline the potential of propensity score modeling to evaluate differences in therapeutic outcomes in patients with cancer in the real-world setting.^31^, ^32^, ^33^ By creating a ‘pseudo-randomized’ population that balances baseline characteristics, this approach helps overcome biases in retrospective studies, providing more reliable estimates of comparative treatment effects.^14^, ^15^, ^16^

Our study has important clinical implications. The higher efficacy of FTD–TPI + bevacizumab in a representative multicenter real-world cohort supports its routine use in patients with mCRC in the late-line setting. Treatment selection in this setting is especially relevant regarding the substantial funnel effect in the advanced-line therapies.^3^ In our study, only one-third of patients received subsequent systemic therapies following disease progression, highlighting the need to prioritize the most effective therapeutic option early in the treatment course. Our study further supports the consistent efficacy of FTD–TPI + bevacizumab across key molecular subgroups, with the potential exception of patients harboring *BRAF*^*V600E*^ mutations. However, given the overall poor outcomes of patients in our study, specifically regarding long-term disease control, these data emphasize the need for improved therapeutic options in the late-line setting, and the necessity to optimize combination treatments and implement an effective treatment continuum over multiple lines of therapy in patients with mCRC to maximize individual clinical benefit. In addition, although the observed improvement in outcomes is statistically significant, its clinical relevance in heavily pretreated patients with mCRC should be interpreted cautiously and weighed against potential treatment burden, including adverse events, quality of life, and time spent receiving medical care.

This study has several limitations that warrant consideration. Firstly, due to the retrospective nature, no central assessment of treatment response was carried out, and responses were categorized according to the interpretations of the local radiologists and treating physicians at each study center. In addition, follow-up procedures including the frequency and timing of restaging were not standardized. Therefore, data captured by individual chart review might be subject to center-specific treatment and surveillance protocols and random missingness of baseline and follow-up data is possible. However, given the routine follow-up practices during active treatment at the participating cancer centers and the structured documentation of clinical information, we consider the integrity and quality of the collected data to be high.

Secondly, individualized clinical decision-making in routine practice may have introduced deviations from trial populations, potentially contributing to the observed differences in outcomes. Furthermore, sequential use of the investigated therapies resulted in considerable longitudinal overlap between treatment groups. In detail, around one-third of patients treated with FTD–TPI ± bevacizumab received subsequent regorafenib therapy and about half of patients treated with regorafenib received subsequent FTD–TPI. Although PFS and DCR were assessed for each treatment line and are thus unaffected, the use of post-progression therapy may have impacted OS estimates and between-group comparisons. This is supported by the results of our sensitivity analysis censoring patients at initiation of the next systemic therapy, which indicated a higher magnitude of OS benefit compared with the primary analysis.

Thirdly, for our comparative analyses, we applied IPTW-adjustment to control for potential confounding by patient- and cancer-specific covariates that could influence both treatment assignment and study outcomes. This approach generates a pseudo-population in which treatment assignment is independent of covariates, thereby mimicking randomization.^15^ Upon inspecting covariable balance after IPTW adjustment, performance of the propensity score was deemed adequate.^14^^,^^16^ However, residual confounding based on known and unknown factors cannot be fully excluded in the framework of this observational study.

Fourthly, our study specifically focused on a comparative effectiveness analysis of FTD–TPI + bevacizumab versus FTD–TPI alone or regorafenib in patients with mCRC, and did not evaluate other late-line therapeutic options, such as molecularly targeted therapies or fruquintinib, which is typically used after progression on FTD–TPI ± bevacizumab or regorafenib.^2^^,^^34^ Therefore, no direct implications for other late-line treatment options, such as fruquintinib, can be drawn, as these fall outside the scope of the present analysis.

Finally, due to non-standardized toxicity reporting across centers, systematic safety and adverse event data were not available, representing an important limitation of this study.

### Conclusion

In summary, our study provides comprehensive data from multiple cancer centers on real-world outcomes of patients with mCRC treated with FTD–TPI + bevacizumab, FTD–TPI monotherapy, and regorafenib. Our data indicate improved efficacy of FTD–TPI + bevacizumab, with consistent findings across key molecular subgroups including patients with *KRAS*^*G12*^ mutations.
