# Supplementary material for: Clinical outcomes of trifluridine/tipiracil plus bevacizumab versus trifluridine/tipiracil or regorafenib in metastatic colorectal cancer: a multicenter cohort study
Source: ESMO Open. 2026 Mar 26;11(4):106907. doi: 10.1016/j.esmoop.2026.106907 (PMC13059099; doi:10.1016/j.esmoop.2026.106907)
Supplement: Supplementary Material [file mmc1.docx]

**Supplementary Appendix**

**Supplementary Table 1: Propensity score model for treatment assignment to FTD–TPI + Bevacizumab vs controls.**

| **Variable** | **Odds Ratio** | **95%CI** | **p** |
| --- | --- | --- | --- |
| **Demographics** |  |  |  |
| Age (per 1 year increase) | 1.02 | 0.99–1.05 | 0.233 |
| Male Sex | 0.80 | 0.49–1.30 | 0.369 |
| Charlson Comorbidity Index (per 1 point increase) | 0.87 | 0.71–1.07 | 0.196 |
| ECOG (0 vs ≥1) | 1.01 | 0.58–1.77 | 0.964 |
| BMI (per 1 point increase) | 0.97 | 0.92–1.02 | 0.231 |
| **Tumor parameters** |  |  |  |
| Right-sided primary | 1.16 | 0.69–1.97 | 0.573 |
| Liver metastasis | 0.91 | 0.53–1.58 | 0.744 |
| RAS-Mutant | 1.04 | 0.63–1.70 | 0.888 |
| Synchronous metastatic | 1.14 | 0.68–1.90 | 0.629 |
| **Treatment data** |  |  |  |
| Treatment line | 0.65 | 0.50–0.86 | 0.003 |
| Study center | 1.78 | 1.55–2.04 | <0.001 |
| **Laboratory parameters** |  |  |  |
| CEA (per 1 unit increase) | 1.00 | 1.00-1.00 | 0.919 |

Abbreviations: BMI: body mass index, CEA: carcinoembryonic antigen, CI: confidence interval, ECOG: Eastern Cooperative Oncology Group performance status, p: p-value.

**Supplementary Table 2: Variables used for multiple imputation with n=25 imputation datasets.**

| **Variable** | **N (complete)** | **N (imputed)** | **Imputation method** |
| --- | --- | --- | --- |
| Age | 508 | 1 | Linear regression |
| BMI | 380 | 129 | Linear regression |
| CEA levels | 326 | 183 | Linear regression |
| Charlson comorbidity index | 409 | 100 | Linear regression |
| DCR | 448 | 61 | Logistic regression |
| Death | 509 | 0 | N/A |
| Disease Progression | 509 | 0 | N/A |
| ECOG | 464 | 45 | Logistic regression |
| Liver metastasis | 509 | 0 | N/A |
| Lung metastasis | 509 | 0 | N/A |
| Male sex | 509 | 0 | N/A |
| OS | 509 | 0 | N/A |
| PFS | 509 | 0 | N/A |
| RAS status | 505 | 4 | Logistic regression |
| Right-sided primary | 509 | 0 | N/A |
| Study center | 509 | 0 | N/A |
| Synchronous metastatic disease | 508 | 1 | Logistic regression |
| Treatment line | 509 | 0 | N/A |
| Treatment type received | 509 | 0 | N/A |

Abbreviations: BMI: body mass index, CEA: carcinoembryonic antigen, ECOG: Eastern Cooperative Oncology Group, DCR: disease control rate, PFS: progression free survival, OS: overall survival.

**Supplementary Table 3: Covariate balance upon IPTW-weighing**

| **Covariable** | **SMD (post-IPTW)** | **SMD (pre-IPTW)** |
| --- | --- | --- |
| CEA | -0.17 | -0.02 |
| Age | -0.17 | 0.19 |
| Right-sided primary | -0.13 | 0.01 |
| Study center | -0.11 | 1.47 |
| CCI | -0.01 | -0.05 |
| Treatment line | 0.00 | -0.51 |
| RAS mutant | 0.00 | -0.02 |
| Male Sex | 0.04 | -0.07 |
| BMI | 0.06 | -0.02 |
| Liver metastasis | 0.08 | 0.07 |
| ECOG | 0.13 | -0.11 |
| Synchronous metastatic | 0.15 | 0.03 |

Abbreviations: BMI: body mass index, CCI: Charlson comorbidity index, CEA: carcinoembryonic antigen, ECOG: Eastern Cooperative Oncology Group.

**Supplementary Table 4: IPTW-adjusted PFS and OS estimates**

|  | **Analysis cohort*** | **Overall** | **FTD–TPI + Bevacizumab** | **Controls** | **P**** |
| --- | --- | --- | --- | --- | --- |
| **PFS**** (median, months) | 509 (100%) | 3.3 | 3.8 | 3.0 | 0.014 |
| **- 3mo (%)** | / | 58.2% | 66.6% | 49.0% | / |
| **- 6mo (%)** | / | 26.0% | 32.6% | 18.7% | / |
| **- 12mo (%)** | / | 8.4% | 10.8% | 5.9% | / |
| **- 24mo (%)** | / | 1.5% | 1.5% | 1.6% | / |
| **OS**** (Median, months) | 509 (100%) | 8.6 | 8.8 | 8.3 | 0.203 |
| **- 3mo (%)** | / | 86.9% | 91.5% | 82.1% | / |
| **- 6mo (%)** | / | 67.5% | 72.3% | 62.5% | / |
| **- 12mo (%)** | / | 40.2% | 42.8% | 37.7% | / |
| **- 24mo (%)** | / | 15.6% | 18.4% | 13.9% | / |

Abbreviations: FTD–TPI: trifluridine/tipiracil, mo: months, OS: overall survival, PFS: progression-free survival.

**Supplementary Table 5: IPTW-adjusted analysis of FTD–TPI + Bevacizumab vs FTD–TPI, and vs regorafenib**

|  | **FTD–TPI + Bevacizumab vs Controls** | **FTD–TPI + Bevacizumab vs FTD–TPI** | **FTD–TPI + Bevacizumab vs Regorafenib** |
| --- | --- | --- | --- |
| **PFS (HR, 95%CI)** | 0.68 (0.51-0.92)  p=0.012 | 0.65 (0.46-0.90)  p=0.009 | 0.71 (0.50-1.02)  p=0.07 |
| **OS (HR, 95%CI)** | 0.80 (95%CI: 0.58-1.12)  p=0.203 | 0.80 (0.56-1.15)  p=0.228 | 0.80 (0.55-1.16)  p=0.246 |
| **DCR (OR, 95% CI)** | 3.35 (95%CI: 1.81-6.20)  p<0.001 | 3.44 (1.79-6.60)  p<0.001 | 3.20 (1.59-6.44)  p=0.001 |
| **ORR (OR, 95%CI)** | 2.25 (95% CI: 0.81–6.25)  p = 0.123 | 2.21 (0.74-6.59)  p=0.156 | 2.32 (0.68-7.93)  p=0.179 |

Abbreviations: CI: confidence interval, DCR: disease control rate, FTD–TPI: trifluridine/tipiracil, HR: hazard ratio, OR: odds ratio, ORR: objective response rate, OS: overall survival, PFS: progression-free survival.

**Supplementary Table 6: Molecular subgroup analysis**

| **Mutational subgroup** | **IPTW-adjusted HR (95%CI) for PFS** | **Interaction p-value (PFS)** | **IPTW-adjusted OR (95%CI) for DCR** | **Interaction p-value (DCR)** | **IPTW-adjusted HR (95%CI) for OS** | **Interaction p-value (OS)** |
| --- | --- | --- | --- | --- | --- | --- |
| Overall (n=509) | 0.68 (0.51-0.92), p=0.012 |  | 3.35 (1.81-6.20), p<0.001 |  | 0.80 (0.55-1.16), p=0.246 |  |
| RAS (n=505) |  | p=0.903 |  | p=0.848 |  | p=0.561 |
| - KRAS mutated (n=276) | 0.66 (0.47-0.94), p=0.019 |  | 3.13 (1.42-6.90), p=0.005 |  | 0.77 (0.48-1.25), p=0.289 |  |
| - WT (n=209) | 0.73 (0.44-1.22), p=0.225 |  | 3.53 (1.33-9.36), p=0.011 |  | 0.88 (0.51-1.53), p=0.655 |  |
| KRAS^G12^ (n=505) |  | p=0.561 |  | p=0.548 |  | p=0.125 |
| - Mutated (n=154) | 0.58 (0.36-0.93), p=0.025 |  | 2.56 (0.91-7.19), p=0.074 |  | 0.54 (0.28-1.03), p=0.063 |  |
| - WT (n=351) | 0.76 (0.51-1.13), p=0.180 |  | 3.80 (1.76-8.21), p=0.001 |  | 1.02 (0.68-1.54), p=0.918 |  |
| PIK3CA (n=170) |  | p=0.900 |  | p=0.670 |  | p=0.423 |
| - Mutated (n=34) | 0.71 (0.36-1.43), p=0.343 |  | 6.93 (0.57-84.64), p=0.130 |  | 1.30 (0.31-5.35), p=0.721 |  |
| - WT (n=136) | 0.55 (0.31-0.99), p=0.049 |  | 3.86 (1.34-11.13), p=0.012 |  | 1.13 (0.53-2.41), p=0.748 |  |
| TP53 (n=143) |  | p=0.204 |  | p=0.169 |  | p=0.198 |
| - Mutated (n=85) | 0.43 (0.21-0.86), p=0.018 |  | 7.30 (2.00-26.61), p=0.003 |  | 0.92 (0.44-1.92), p=0.824 |  |
| - WT (n=58) | 0.78 (0.40-1.50), p=0.453 |  | 1.37 (0.18-10.26), p=0.758 |  | 0.63 (0.15-2.78), p=0.546 |  |
| BRAF^V600E^ (n=421) |  | p=0.020 |  | p=0.421 |  | p=0.357 |
| - Mutated (n=22) | 1.28 (0.54-3.05), p=0.576 |  | 1.43 (0.11-19.21), p=0.787 |  | 0.69 (0.16-2.94), 0.618 |  |
| - WT (n=399) | 0.56 (0.40-0.79), p=0.001 |  | 4.20 (2.13-8.29), p<0.001 |  | 0.77 (0.50-1.18), p=0.231 |  |

Abbreviations: HR: hazard ratio, DCR: disease control rate, IPTW: inverse probability of treatment weight, OR: odds ratio.

**Supplementary Table 7: Overview of efficacy outcomes in landmark phase III trials and outcomes in the present study**

| **Outcome** | **RECOURSE (FTD–TPI-arm)** | **CORRECT**  **(Regorafenib arm)** | **SUNLIGHT** | | **Real-world multi-center cohort** | | |
| --- | --- | --- | --- | --- | --- | --- | --- |
|  |  |  | **FTD–TPI + Bevacizumab** | **FTD–TPI** | **FTD–TPI + Bevacizumab** | **FTD–TPI** | **Regorafenib** |
| **DCR** | 44% | 41% | 69.5% | 41.9% | 51.3% | 22.6% | 23.6% |
| **ORR** | 1.6% | 1.0% | 6.1% | 1.2% | 9.7% | 5.1% | 4.7% |
| **PFS (months)** | 2.0 (1.9-2.1) | 1.9 (1.6-3.9) | 5.6 (4.5-5.9) | 2.4 (2.1-3.2) | 4.1 (3.5-5.3) | 3.0 (2.7-3.2) | 3.0 (2.5-3.4) |
| **6-month** | / | / | 43% | 16% | 34.8% | 17.5% | 17.2% |
| **12-month** | / | / | 16% | 1% | 9.0% | 2.6% | 7.3% |
| **24-month** | / | / | / | / | 3.4% | 0% | 3.1% |
| **HR (PFS) vs controls*** | 0.48 (0.38-0.60) | 0.49 (0.42-0.58) | 0.44 (0.36-0.54) | Ref | 0.68 (0.51-0.92) | Ref | Ref |
| **KRAS-wt** | 0.48 (0.38-0.60) | 0.48 (0.36-0.62) | 0.29 (0.20-0.42) | Ref | 0.74 (0.53-1.04) | Ref | Ref |
| **KRAS-mut** | 0.49 (0.39-0.61) | 0.53 (0.43-0.65) | 0.51 (0.41-0.64) | Ref | 0.73 (0.55-0.96) | Ref | Ref |
| **OS** | 7.1 (6.5-7.8) | 6.4 (3.6-11.8) | 10.8 (9.6-12.1) | 7.2 (6.3-8.5) | 8.7 (7.7-10.2) | 8.3 (6.5-9.4) | 8.1 (6.8-10.6) |
| **6-month** |  | 52.5% | 77% | 61% | 71.0% | 61.0% | 62.6% |
| **12-month** | 27% | 24.3% | 43% | 30% | 38.2% | 35.5% | 37.3% |
| **24-month** | / | / | / | / | 15.9% | 10.0% | 14.8% |
| **HR (OS) vs controls*** | 0.68 (0.58-0.81) | 0.77 (0.64-0.94) | 0.61 (0.49-0.77) | Ref | 0.80 (0.58-1.12) | Ref | Ref |
| **KRAS-wt** | 0.58 (0.45-0.74) | 0.65 (0.48-0.90) | 0.64 (0.43-0.96) | Ref | 0.88 (0.51-1.53) | Ref | Ref |
| **KRAS-mut** | 0.80 (0.63-1.02) | 0.87 (0.67-1.12) | 0.62 (0.48-0.81) | Ref | 0.77 (0.48-1.25) | Ref | Ref |

*Controls represent placebo arm in the RECOURSE- and CORRECT-trial, FTD–TPI monotherapy in the sunlight trial, and FTD–TPI- or regorafenib monotherapy in this cohort study. IPTW-adjusted HRs reported for the present cohort study.

**Supplementary Figure 1: Histogram of propensity score**

**Supplementary Figure 2: Kernell Density plot of propensity scores by treatment groups**

**Supplementary Figure 3: IPTW-adjusted hazard of disease progression over time.**

Hazard curved predicted from a flexible parametric survival model.

**Supplementary Figure 4: IPTW-adjusted predicted probability of PFS according to treatment assignment.**

Survival curves predicted from a flexible parametric survival model.

***Supplementary Figure 5: IPTW-adjusted hazard of death over time.***

Hazard curves predicted from a flexible parametric survival model.

**Supplementary Figure 6: IPTW-adjusted predicted probability of OS according to treatment assignment.**

Survival curves predicted from a flexible parametric survival model.

**Supplementary Figure 7: IPTW-adjusted Kaplan Maier estimates of PFS according to molecular subgroups**
